# Supplementary material for: Immunolocalization of Two Neurotrophins, NGF and BDNF, in the Pancreas of the South American Sea Lion Otaria flavescens and Bottlenose Dolphin Tursiops truncatus
Source: Animals (Basel). 2024 Aug 13;14(16):2336. doi: 10.3390/ani14162336 (PMC11350702; doi:10.3390/ani14162336)
Supplement: Supplementary file 1 [file animals-14-02336-s001.zip › animals-3143398-supplementary.pdf]

Immunolocalization of two neurotrophins, NGF and BDNF, in the pancreas of the South American Sea Lion *Otaria flavescens* and Bottlenose Dolphin *Tursiops truncatus*

Claudia Gatta [1], Luigi Avallone[1], Anna Costagliola[1], Paola Scocco[2], Livia D'Angelo[1], Paolo de Girolamo[1], Elena De Felice[2,*]

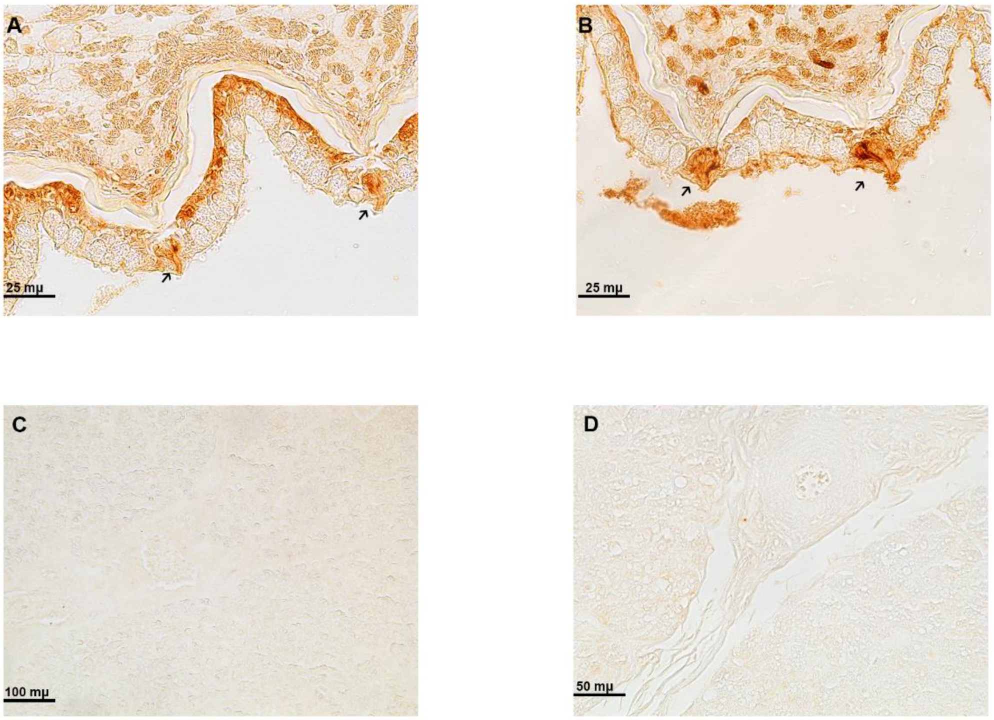

Figure S1. Immunohistochemistry positive and negative controls. Positive NGF control in zebrafish taste buds (indicated with arrows) **A**; positive BDNF control in zebrafish taste buds (indicated with arrows) **B**; negative pancreas control *Otaria flavescens* **C**; negative pancreas control *Tursiops truncatus* **D**.
